# Dental Anatomy—Human and Comparative

**Published:** 1890-02

**Authors:** 


DENTAL ANATOMY—HUMAN AND COMPARATIVE.—By Charles S. Tomes, M. A., F. R. S., with 212 illustrations. Third edition. Philadelphia: P. Blakiston, Son & Co., 1890.

This new edition of a standard work, gives a more extended outline of the important facts of odontology than the former editions. Hypotheses as to the genesis of teeth, &c., have either been omitted, or passed by with brief mention, for the alleged reason that they have not as yet so established themselves as to meet with general acceptance.

Due acknowledgement is given by the author to the odontographies of Prof. Owen and of Giebel; to Prof. Flower's lectures on odontology and his articles in the Encyclopædia Britanica; to Dr. Worthman's article on the teeth in the American System of Dentistry, and to the contributions of Profs. Cope and Marsh in regard to the extinct mammalian fauna of America, of all of which he has made free use.

This third edition is a valuable contribution to dental literature, and will prove a useful adjunct in the study of the human and comparative anatomy of the teeth.

